# First Discovery of the North American Leaf-Mining Moth *Chrysaster ostensackenella* (Lepidoptera: Gracillariidae) in Russia: The Genetic Diversity of a Novel Pest in Invaded vs. Native Range

**DOI:** 10.3390/insects14070642

**Published:** 2023-07-15

**Authors:** Natalia I. Kirichenko, Nina A. Kolyada, Stanislav Gomboc

**Affiliations:** 1 Sukachev Institute of Forest, Siberian Branch of the Russian Academy of Sciences, Federal Research Center «Krasnoyarsk Science Center SB RAS», Akademgorodok 50/28, 660036 Krasnoyarsk, Russia; 2 Institute of Ecology & Geography, Siberian Federal University, Svobobny pr. 79, 660041 Krasnoyarsk, Russia; 3 Federal Scientific Center of the East Asia Terrestrial Biodiversity, Far Eastern Branch of the Russian Academy of Sciences, 100-Let Vladivostoku Ave. 159, 690022 Vladivostok, Russia; kolyada18@rambler.ru; 4 Independent Researcher, Gančani 110, 9231 Beltinci, Slovenia; stanislav.gomboc@siol.net

**Keywords:** alien species, black locust, first record, gracillariid moth, invasion, Russian Far East

## Abstract

**Simple Summary:**

The leaf-mining moth *Chrysaster ostensackenella* (Fitch, 1859) (Lepidoptera: Gracillariidae) is the North American species that damages the leaves of *Robinia* spp. (Fabaceae). Recently, it was detected on the introduced North American black locust *Robinia pseudoacacia* (Fabaceae) in East Asia (China, South Korea, Japan) and Europe (Italy). In July 2022, *Ch. ostensackenella* was for the first time found on the territory of Russia, in Primorsky Krai. We studied the bionomics of this invasive moth and highlighted the characteristics of leaf damage by which the presence of *Ch. ostensackenella* can be detected early. Furthermore, we DNA barcoded three moth specimens from Primorsky Krai and analyzed them against DNA barcodes of *Ch. ostensackenella* from invaded countries in Europe (Italy) and East Asia (South Korea and Japan) and the native range (North America). Overall, our findings suggest that *Ch. ostensackenella* distributed to East Asia from the USA directly, not through Europe, whereas to the Russian Far East, this invasive moth spread from early invaded East Asian countries, in particularly Japan.

**Abstract:**

Here, we report the first detection of the North American leaf-mining moth *Chrysaster ostensackenella* (Fitch, 1859) (Lepidoptera: Gracillariidae) on North American black locust *Robinia pseudoacacia* (Fabaceae) in Primorsky Krai (the Russian Far East) in July 2022. Overall, six moths were reared from the leaf mines and identified based on adult morphology (forewing pattern and male genitalia) and three of them were DNA barcoding. Description of the leaf mines that allowed us to distinguish the damage of *Ch. ostensackenella* from other gracillariids associated with *R. pseudoacacia* is provided. The phylogeographic analysis comparing the DNA barcodes from Russia with those from other invaded countries in Europe (Italy) and East Asia (South Korea and Japan) and from the native range (North America) was performed. Intraspecific genetic diversity reached 3.29%. Altogether, 10 haplotypes were revealed among 21 studied specimens in the Holarctic. The detection of one haplotype common for Japan and the USA (North Carolina) suggests that the invasion to East Asia could have happened from the USA directly, rather than through Europe. A shared haplotype defined for Japan and the Russian Far East points at a possible moth species’ spread to Primorsky Krai from earlier invaded Hokkaido. Further distribution of *Ch. ostensackenella* in East Asia and Europe is expected, bearing in mind the wide planting of *R. pseudoacacia* in these continents. Furthermore, an accidental introduction of the moth to the Southern Hemisphere, where black locust was introduced, is not ruled out.

## 1. Introduction

Leaf-mining insects are among the most common pests affecting ornamental woody plants and crops worldwide [1,2,3,4]. Their larvae make cavities of different shapes and sizes inside leaf tissues, which are called leaf mines [5,6]. During outbreaks, such insects cause leaf discoloration or browning (due to eating out leaf tissues in the mines) leading to drying and the premature falling of leaves [2,7,8,9].

Despite their small size and limited flying capabilities, the adults of leaf miners can disperse over long distances via wind currents, acting as air plankton, or by hitchhiking on various human transport vehicles (land, air, or water) [10,11]. Additionally, adult and immature stages (eggs, larvae, and pupae) can be transported between regions and continents with host plants, particularly those used for planting, cut flowers, etc. [2,10,12]. In recent decades, with the rise of international trade, some leaf-mining insects have become invasive, expanding beyond their original ranges and causing significant damage to woody plants in new regions and countries [2,13]. In Southern Russia alone, in the last two decades seven alien leaf-mining gracillariids, *Macrosaccus robiniella* (Clemens, 1859); *Parectopa robiniella* Clemens, 1863; *Phyllonorycter issikii* (Kumata, 1963); *Ph. leucographella* (Zeller, 1850); *Ph. platani* (Staudinger, 1870); *Phyllocnistis citrella* Stainton, 1856; and *Cameraria ohridella* (Deschka & Dimić, 1986), have been detected and identified as significant pests, notably attacking some woody plants [14].

The family Gracillariidae comprises a diverse group of leaf-mining moths known for their feeding habits on a wide range of woody and herbaceous plants [4,15]. Among them, several alien species have become pests on woody plants with ornamental value.

Black locust *Robinia pseudoacacia* L. (Fabaceae) is a North American plant which was introduced to the European continent in the 17th century and used for various purposes [16,17]. Over time, it was planted in the European part of Russia and introduced to arboreta in the southern part of the Russian Far East, specifically in Primorsky Krai [18,19].

A few decades ago, two North American leaf-mining gracillariids, *Parectopa robiniella* and *Macrosaccus robiniella*, trophically associated with *R. pseudoacacia*, penetrated the European continent and distributed to the European part of Russia, causing pronounced damage to their host plant [2,20,21]. Another leaf-mining gracillariid known to infest *R. pseudoacacia* in North America is *Chrysaster ostensackenella* (Fitch, 1859) [15,22]. In recent years, the species has been documented in China in 2015 [23,24], South Korea in 2017 [25], Japan in 2021 [26], and Europe (so far only in Italy) in 2022 [27].

Here, we report the first finding of *Ch. ostensackenella* in Russia, in Primorsky Krai, provide data on the species bionomics, and compare the DNA barcodes of the Russian specimens with those from the native (USA and Canada) and invaded range (Italy, South Korea, Japan). Additionally, we provide a detailed description of the *Ch. ostensackenella* leaf mine, emphasizing diagnostic characteristics that enable differentiation from two other invasive gracillariid species from North America, *P. robiniella* and *M. robiniella*, associated with *R. pseudoacacia*.

## 2. Materials and Methods

### 2.1. Study Area

**Figure 1:**
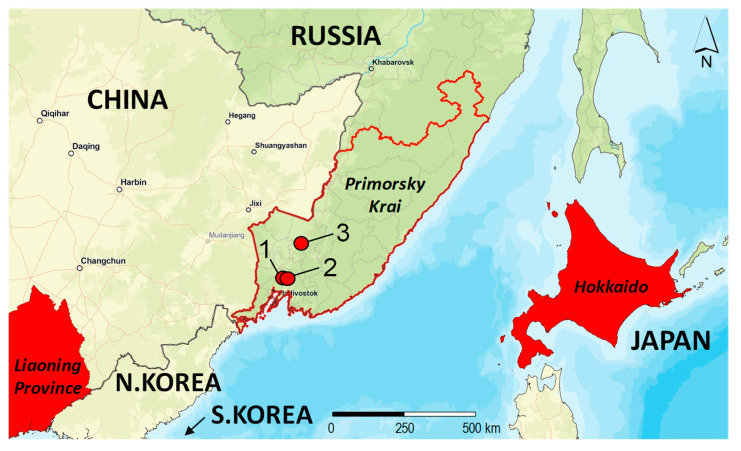
The localities in Primorsky Krai (the Russian Far East), where leaves with the mines of *Chrysaster ostensackenella* were sampled on *Robinia pseudoacacia* in 2022, and the proximity of the territories in China and Japan where the moth was detected in 2015 and 2021, respectively. Primorsky Krai: 1—Gornotaejnoe, arboretum of MTS; 2—Gornotaejnoe, village plantings; 3—planting next to Khorol village. The map was produced using ArcGIS Pro software, version 3.1 [29].

The visual inspections of *Robinia pseudoacacia* were conducted in three localities in Primorsky Krai (Russian Far East): the arboretum of the Mountain-Taiga Station (MTS) in Gornotaejnoe village (43.6942 N, 132.1535 E), Gornotaejnoe village (43.6955 N, 132.1566 E), and the surrounding area of the village Khorol (44.4031 N, 132.1180 E) (Figure 1). The distance between Gornotaejnoe and Khorol was approximately 71.2 km in a straight line, or about 96 km when following the roads. *R. pseudoacacia* was introduced to the Russian Far East at the beginning of the 20th century [28].

The maps of East Asia, the world, and the enlargement of the invaded regions were produced using the WGS 1984 coordinate system and ESRI ArcGIS Pro 3.1 software [29]. Layers from publicly available web services in ArcGIS Online were utilized within ArcGIS Pro for creating the map, which were accessible as geographic web services.

### 2.2. Sampling, Rearing, Photographing

In each of the sampled localities, between 5 to 10 trees ranging in height from 3 to 9 m were examined. In the period from July 10th to August 8th, 2 to 4 low branches were inspected on each tree to determine the presence of roundish mines on the leaves. A total of 20 leaves with the mines containing late instar larvae were collected and transported to the laboratory at MTS (Gornotaejnoe).

For further analysis, mined leaves were individually placed in 50-mL zip-lock plastic bags along with a wiping tissue to control excessive humidity. The bags were monitored every second day to document adult emergence. One male was dissected, and the genitalia slide prepared following Robinson [30]. The photographs leaf mines, moths, and genitalia were taken with a Xiaomi 11 Lite smartphone digital camera (Beijing, China, Xiaomi Corporation) in conjunction with an Olympus SZ51 stereomicroscope (Tokyo, Japan, Olympus Corporation) and a Biotrinocular panchromatic 2002 microscope EUM-2000 (Chongqing, China, Chongqing Optec Instrument Co., Ltd.). The images were processed in Adobe Photoshop CS6 software. The pinned moths and herbarized leaves are stored in the collection of the first author at the Sukachev Institute of Forest SB RAS in Krasnoyarsk.

### 2.3. DNA Barcoding

To genetically characterize *Ch. ostensackenella*, three adult specimens reared from leaf mines on *R. pseudoacacia* in Primorsky Krai were DNA barcoded. For DNA extraction, the abdomens of two female specimens were used, while in the case of the male specimen, which was used for genital dissection, two hind legs were utilized.

The DNA barcoding fragment of the mitochondrial cytochrome oxidase I gene (mtDNA COI, 658 bp) was sequenced using the primer set C_LepFolF/C_LepFolR following the standard protocol [31]. The analysis was performed at the Canadian Center for DNA barcoding (CCDB) at the University of Guelph (Canada). The sequences, trace files, biogeographic data and images of insect vouchers were deposited in the Barcode of Life Data Systems (BOLD) [32] and GenBank. The data are publicly accessible in BOLD using the link dx.doi.org/10.5883/DS-CHRYSORU (16 May 2023).

Overall, 18 borrowed sequences of *Ch. ostensackenella* were used to explore intraspecific divergence and haplotype diversity. Their specimen data are listed in Table 1.

Among them, nine sequences were publicly available in BOLD: eight specimens originated from the native species range, North America, i.e., seven from the USA (three from North Carolina, three from Tennessee, one from Virginia), one from Canada (Ontario), and one was from the invaded place in Europe (Italy). Eight sequences published as a new species from Japan [26] were retrieved from GenBank; originally, they were not deposited in BOLD (Table 1). Finally, one DNA barcode unique for 7 specimens sequenced from South Korea was published in the paper [25], without submitting to GenBank or BOLD (therefore no GenBank accession number nor Process ID is available) (Table 1). The DNA sequence of the Korean specimen was directly borrowed from the paper and converted to FASTA format for the analysis.

The Barcode Index Number (BIN) of the analyzed specimens was retrieved from BOLD [33]. The alignment was performed in BioEdit 7.2.5 [34]. A maximum likelihood tree was built in MEGA X [35] using the maximum likelihood method, the Kimura two-parameter model, and a bootstrap method (1000 iterations). Intraspecific distances were estimated using the same approaches. The minimal intraspecific distances were calculated for the specimens from different geographic regions, and minimal and maximal values were estimated for geographic populations represented by more than two individuals per region. The DNA barcode of the related leaf-mining moth *Chrysaster hagicola* Kumata, 1961 from the Russian Far East, obtained in our early study, was used to root the phylogenetic tree (Table 1). The median-joining haplotype network was constructed using the program Pop ART (version 1.7 for Windows) implementing a statistical parsimony algorithm [36].

## 3. Results

### 3.1. Novel Record and the Species Biology

*Chrysaster ostensackenella* (Fitch, 1859)

*Synonums: Argyromiges ostensackenella* Fitch, 1859; *Lithocolletis ostensackenella* Chambers, 1871; *Lithocolletis ornatella* Chambers, 1871.

*Material examined*. Russia: Primorsky Krai, Ussurijsk Okrug, Gornotaejnoe village, arboretum of MTS, 18–25 July 2022 leaf mines with larvae coll., *Robinia pseudoacacia* (host plant), 30 July–15 August 2022 adults emerged (em.), 1♂, 2♀, including two DNA-barcoded specimens (1♂ and 1♀) (Sample ID: NK1931, GenBank accession number OR178285; NK1929, OR178280), N.A. Kolyada coll.; ibidem, but planting in Gornotaejnoe village, 24 July 2022 leaf mine with larva coll., 1 August 2022 em., 1♀, N.A. Kolyada coll.; ibidem, but in the Khorolsky District, with tree planting near Khorol village, 8 July 2022 leaf mines with larvae coll., 18 August 2022 em., 2♀, including one DNA-barcoded specimen (1♀) (NK1930, OR178279), N.A. Kolyada coll.

**Figure 2:**
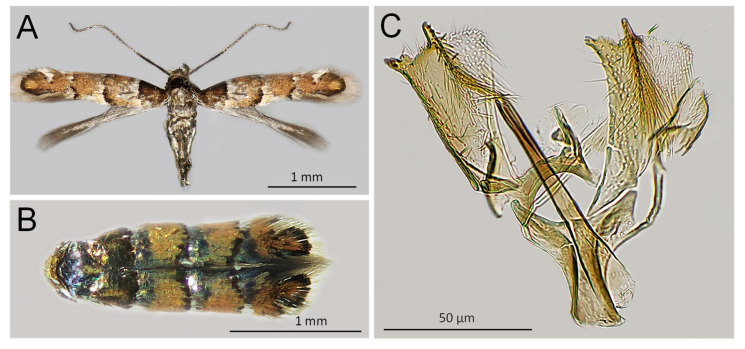
*Chrysaster ostensackenella*: male (**A**,**B**) and its genitalia (**C**); Sample ID: NK1931, Mountain-Taiga Station, Gornotaejnoe, Primorsky Krai, Russia. Photo N. Kirichenko.

*Adults* (Figure 2). The morphology of the adults collected in the Russian Far East is consistent with the species description [24,25]. Therefore, the information on the moth morphology is not repeated here. The body length of the specimens from the Russian Far East was 3.1 ± 0.05 (♂) and 3.3 ± 0.04 (♀) mm; the wingspan was 4.3 ± 0.05 (♂) and 4.4 ± 0.04 (♀) mm.

**Figure 3:**
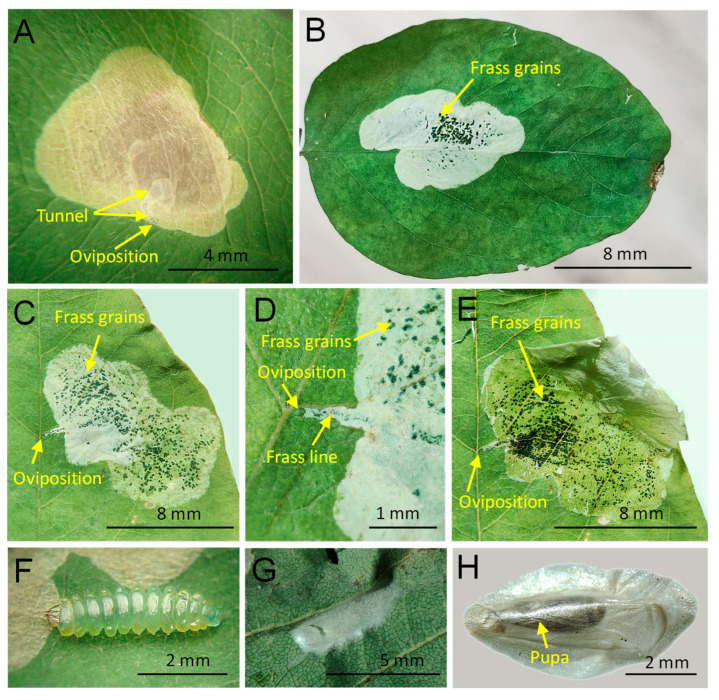
Biology of *Chrysaster ostensackenella.* (**A**) Freshly collected leaf mine on the upper side of the leaf compounds of *Robinia pseudoacacia*; (**B**,**C**) herbarized leaves with the mines with a visible short tunnel proceeding the blotch mine (**C**–**E**); (**F**) the larva dissected from the leaf mine; (**G**) abandoned white silky cocoon on the lower side of the leaf compound; (**H**) the cocoon with the pupa inside. Mountain-Taiga Station, Gornotaejnoe, Primorsky Krai, Russia. Photo N. Kolyada and N. Kirichenko.

*Biology* (Figure 3). In the Russian Far East, we observed relatively large yellowish blotch mines on the upper side of the leaf compounds, with a light brown area in the central part of the mine (Figure 3A). However, it is important to note that herbarized mines exhibited a color change from yellow to white, and the frass became more visible, even in gently pressed leaves (Figure 3B). 

In 10 studied mines, the oviposition site was typically located near the main vein or between the main vein and a secondary vein. From the oviposition site, a short tunnel (1.5–2 mm in length) followed a secondary vein before suddenly widening into a blotch (Figure 3C,D). In some cases, the short tunnel was overrun by the blotch, but it was still detectable by its slightly lighter color (whitish) when compared to the main colors of the mine (Figure 3A).

In the Russian Far East, old mines occupied from 10 to 30% of leaf compound square, whereas in China over 50% [24]. The mines were primarily located next to the main vein on one of the halves of the leaf compound. They rarely extended through the main vein to the other half of the leaf compound. Within the tunnel, the frass was deposited in a dotted central line (Figure 3C,D). In the blotch mines, black grains of frass were scattered throughout the mine, with the highest concentration in the central part of the mine (Figure 3E).

Based on our observations in the Russian Far East, each mine contained a single larva. The late instar larva was yellow-greenish in color and measured up to 4 mm in length (Figure 3F). Prior to pupation, the larva created a semicircular cut in the upper epidermis near the margin of the mine for escaping from the mine. Pupation occurred outside the leaf mine, specifically on the lower side of the leaf compound, along a secondary vein, inside a white tough cocoon (Figure 3G,H). The pupation site was noticeable from the upper side of the leaf by the slightly deformed or bent leaf surface along the secondary vein. On the contrary, in North America the pupation was documented inside the leaf mine [22]. In the Russian Far East, a few larvae pupated on the walls of the plastic bags in which the mined leaves were kept. The pupa was dark brown in color (Figure 3H). Under laboratory conditions with a temperature of +24 °C and 45% humidity, pupal development took up to six days.

The number of generations that the species may develop in Primorsky Krai is currently unclear. Based on our observations, it may have two generations in the studied region. In China, *Ch. ostensackenella* develops up to four generations, gradually increasing damage to *Robinia* leaves throughout the season [24]. It is important to note that the number of generations may vary depending on climatic conditions and weather [24].

**Figure 4:**
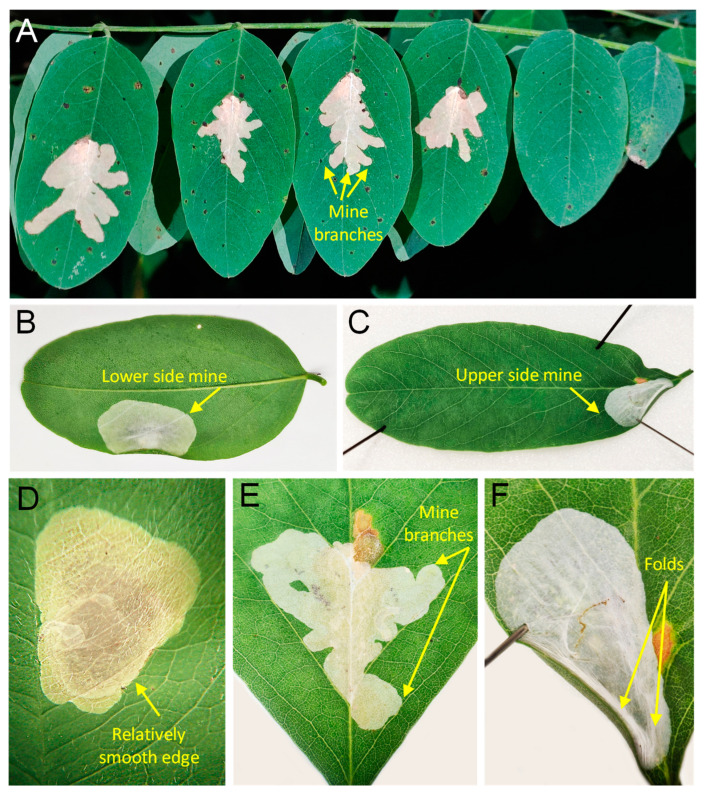
Leaf mines of three North American *Robinia*-feeding gracillariids in Eurasia: (**A**,**E**) branched blotch mines of *Parectopa robiniella* situated over the midrib, Slovenia, July 2022; (**B**,**C**,**F**) lower and upper side blotch (tentiform) mines of *Macrosaccus robiniella*, ibidem; (**D**) the blotch mine of *Chrysaster ostensackenella*, Russia, Promorsky Krai, August 2022. Photo N. Kirichenko, S. Gomboc and N. Kolyada.

*Mine diagnosis* (Figure 4). The characteristics of the blotch mines of *Ch. ostensackenella*, *P. robiniella*, and *M. robiniella* can help distinguish between these species in the field.

Compared to the blotch mine of *P. robiniella*, the mine of *Ch. ostensackenella* is not strictly associated with the main vein (i.e., lays and stretches above it) and has a relatively smooth edge (vs. the notably branched mine of the former species) (Figure 4A,D,E). The blotch mine of *Ch. ostensackenella* begins with a short tunnel. In contrast, the blotch mine of *P. robiniella* does not have a preceding tunnel.

The mines of *Ch. ostensackenella* and *P. robiniella* are located on the upper side of leaf compounds and can be distinguished from the mine of *M. robiniella*, which is mostly found on the lower side of the leaf compound (Figure 4B). In Slovenia, however, upper-side mines of *M. robiniella* have been observed on *R. pseudoacacia* (Figure 4C)*,* although they were much less frequent compared to lower-side mines. These upper-side mines in Slovenia were documented in several localities in 2022 [37].

The mine of *M. robiniella* is always white with slightly folded epidermis covering the mine, giving it a somewhat volumetric appearance (Figure 4F). Additionally, the mine of *M. robiniella* is typically found on one-half of the leaf compound and is not strictly associated with the main vein, like the mine of *P. robiniella*.

*Ch. ostensackenella* and *P. robiniella* pupate outside of the leaf mine, while *M. robiniella* larvae pupate inside the mine. In high-density populations of *M. robiniella*, leaf mines on leaf compounds can merge, and multiple pupae can be found within a single mine.

*Host plants. Ch. ostensackenella* is a monophagous species feeding exclusively on the plants from the genus *Robinia* (Fabaceae). Most *Robinia* spp. are native to North America, but over the past few centuries, one species, *R. pseudoacacia*, has been introduced and become naturalized in various regions of Europe, Asia, and even in the Southern Hemisphere, and presently is considered as an invasive plant in some areas [38,39,40,41]. In the native range (North America), larvae of *Ch. ostensackenella* feed on *Robinia hispida* L., *R. neomexicana* A.Gray, *R. pseudoacacia*, and *R. viscosa* Michx. ex Vent. [15]. In the Russian Far East, the mines were recorded only on *R. pseudoacacia.*

*Damage level.* In July 2022, the alien species did not cause significant damage to *Robinia pseudoacacia* in the studied localities in Primorsky Krai. Only a small percentage, ranging from 1 to 5% of the examined leaves (per 100 leaves), showed signs of mining activity. Typically, each infested leaf carried only one mine, and occasionally a leaf had two mines. In contrast, in China, the species was for the first time documented in 2015 and had already caused substantial damage to *R. pseudoacacia* (up to 90% of infested leaves on the trees) and led to premature leaf fall [24].

### 3.2. Genetic Data in Invaded vs. Native Rage

**Figure 5:**
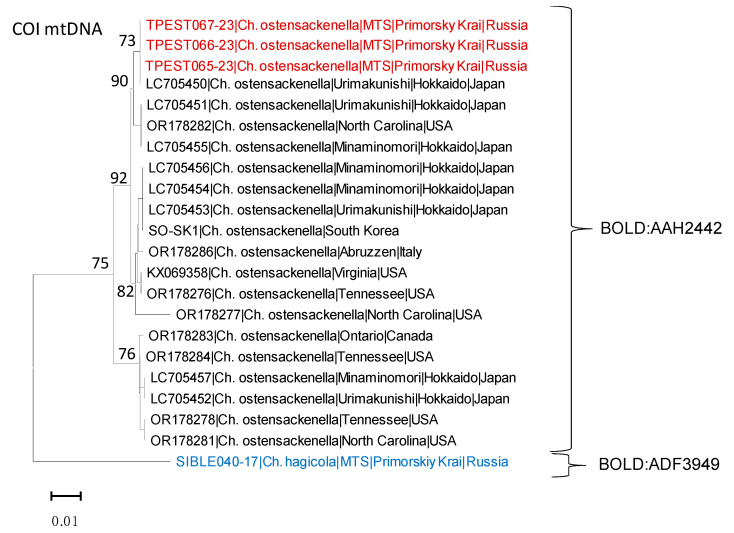
Maximum likelihood COI tree showing the relatedness of *Chrysaster ostensackenella* from Russia (indicated by red color) with the specimens from the USA (native range), Italy, South Korea, and Japan (invaded range). Each specimen is indicated by a GenBank accession number, species name, region, and country. Next to the clusters, BIN numbers are provided. MTS—Mountain-Taiga Station. *Ch. hagicola* (indicated in blue color) was used to root the tree.

The DNA barcoding analysis of three adults from the Russian Far East confirmed their identification as *Ch. ostensackenella* with a high confidence level. The Russian specimens were found to be 100% identical to a specimen from Japan (Urimakunishi) indicated in the GenBank database under the accession number LC705450 (Figure 5, Table 2). The DNA barcodes of the Russian specimens were 100% identical to each other; in BOLD, they matched the unique BIN of the species (BOLD:AAH2442).

Comparing the Russian specimens with those from other geographic regions, the highest similarity was observed with specimens from the USA, specifically from North Carolina (GenBank accession number: OR178282), with a minimal divergence of 0.47%. This was followed by specimens from South Korea (0.63%) and other locations in the USA, namely Tennessee (OR178276) and Virginia (KX069358), both showing a minimal divergence of 0.63% (Figure 2, Table 2). The specimens from Russia showed 0.95% divergence when compared to that from Italy, while the divergence between the Russian and Canadian specimens reached 2.08% (Figure 5, Table 2).

Among eight specimens from Japan, the genetic divergence varied from 0 to 2.08% (Table 2). In this country, the highest genetic divergence (2.08%) was observed between three specimens: two from Minaminomori (GenBank accession numbers: LC705455 and LC705457) and from Urimakunishi from Japan, Hokkaido (LC705451) (Figure 5, Table 2).

Interestingly, one specimen from Japan, Urimakunishi (LC705451) was found to be 100% identical to the specimen from North Carolina (OR178282), while three specimens from Japan, Minaminomori (LC705456 and LC705454), and Urimakunishi (LC705453) showed complete identity to the analyzed DNA barcode from South Korea (Figure 5, Table 2).

For the Italian specimen (OR178286) the nearest neighbors were those from Japan (three specimens), South Korea, and the USA (Tennessee, Virginia), with 0.32% minimal divergence in each case (Table 2).

In the native range (North America), the most variable genetic distances were observed between specimens from North Carolina and Tennessee, from 0% to 3.29% (Table 2). This substantial intra-specific genetic divergence suggests a rich haplotype diversity within this species, and it raises the possibility of presence of a cryptic sibling species.

**Figure 6:**
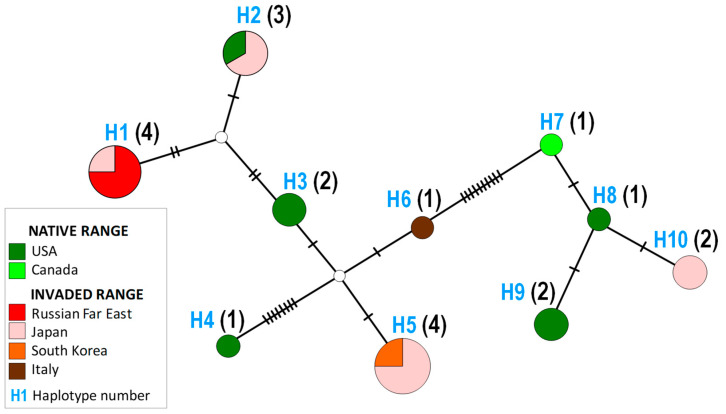
The haplotype network of *Chrysaster ostensackenella* in the Holarctic. The haplotypes (H1–H10) are represented by circles. The number of individuals belonging to each haplotype is indicated in parentheses next to the haplotype. The haplotypes are connected with a 95% confidence level. The colored sectors represent the countries or regions where the haplotypes were found. The vertical strokes on the connection lines and two small empty circles represent hypothetical haplotypes not observed in the study. The geographical distribution of all haplotypes is reported in Table 3.

Among the limited number of studied specimens (overall 21 sequences from native and invaded regions and countries), a total of 10 haplotypes were identified (Tajima’s D = 4707.27, pD ≥ 4907.27 = 0) (Figure 5, Table 3). Interestingly, no clear geographic pattern was observed in the haplotype network (Figure 6).

The most common haplotypes were H1 and H5, which were detected in the invaded range (Russian Far East, Japan, and South Korea) (Figure 6, Table 3). Haplotype H2 was the only one shared between the invaded country (Japan: Hokkaido—Minaminomori and Urimakunishi) and the native range (USA: North Carolina) (Figure 5, Table 3). Two unique haplotypes, H6 and H10, were found in the invaded countries of Italy and Japan, respectively (Figure 5, Table 3). The remaining five haplotypes, H3, H4, H7, H8, and H9, were identified exclusively among the specimens from the native range of *Ch. ostensackenella* in the USA and Canada (Figure 6, Table 3).

The minimal divergence between two representatives of the species *Chrysaster*, the North American *Ch. ostensackenella* and East Asian *Ch. hagicola*, was 8.09%.

## 4. Discussion

**Figure 7:**
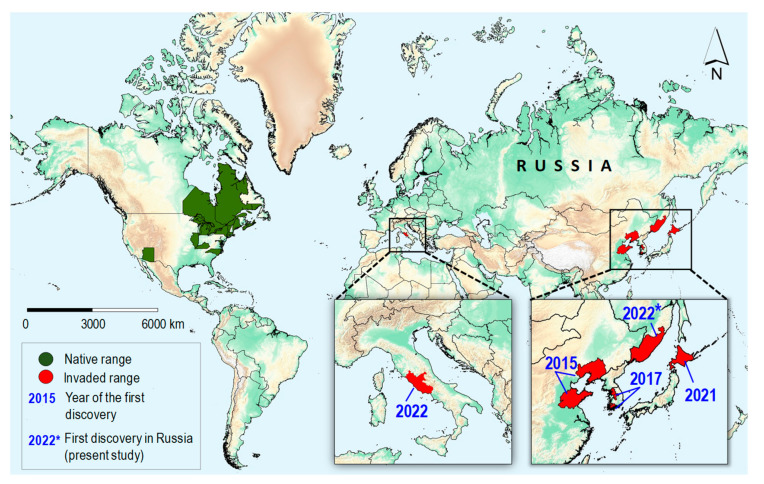
Modern range of *Chrysaster ostensackenella* in the Holarctic. The native range of the species as determined from data gathered in [15] is indicated. The year of the species discovery beyond native range: 2015 in China (Shandong, Liaoning Provinces) [23,24], 2017 in South Korea (Cheongju-si, Seongnam-si, Yeosu-si, Seoul) [25], 2021 in Japan (Hokkaido: Minaminomori, Urimakunishi Prefectures) [26], 2022 in Italy (Monte Terminillo) [27], and 2022 * in Russia (Primorsky Krai: Gornotaejnoe, Khorol villages) (present paper). The maps were produced using ArcGIS Pro software [29].

In the last seven years, the North American *Ch. ostensackenella* has been recorded both in Europe and Asia, thousands of kilometers away from the moth’s native range (Figure 7), puzzling understanding its invasion pattern.

Our study revealed the presence of at least one haplotype common for Japan (invaded country) and the USA, specifically North Carolina (native range), suggesting a potential distribution of the species to East Asia from the USA directly, not through Europe. Interestingly, the specimens from the Russian Far East did not match any haplotype from North America, nor did they correspond to the haplotype found in Europe (Italy) or South Korea. However, they did match a haplotype from Japan, which, however, has not yet been discovered in North America.

It is important to note that the genetic dataset analyzed in our study was relatively small, consisting of 21 specimens from both the native and invaded regions. This limited sampling cannot capture all possible haplotypes, considering the potentially high haplotype diversity in North America, where the highest interspecific divergence was found reaching 3.29% in North Carolina (USA).

The origin of *Ch. ostensackenella* in the Russian Far East remains uncertain. However, based on the available evidence, it is plausible to speculate that the moth reached the region through a phenomenon called the “bridgehead effect”, i.e., the dispersal from an established range to a new area through intermediate locations [42]. This hypothesis suggests that *Ch. ostensackenella* spread to Primorsky Krai from early invaded East Asian countries: China, Korea, and/or Japan.

In China and Korea, the species was first documented only 5–7 years before its initial detection in the Russian Far East in 2022 (Figure 7). This period suggests that the moth had time to establish populations in these countries and subsequently expand its range toward the neighboring territory of the Russian Far East.

Unfortunately, the lack of DNA barcoding data from China does not allow us to test this hypothesis. The genetic analysis conducted on specimens from the Russian Far East revealed that they did not match the single haplotype identified in South Korea. However, the study did uncover a shared haplotype between Japan (eight specimens) and the Russian Far East (three specimens). *Ch. ostensackenella* was first detected in Japan in 2021 (Figure 7), in at least two locations in Hokkaido. The species had already caused noticeable damage to *R. pseudoacacia* in Hokkaido, indicating that it had likely invaded Japan several years prior to its formal documentation in 2021 (Figure 7).

Thus, considering the relatively close proximity (about 400 km by sea) between Primorsky Krai and Hokkaido, it is conceivable that *Ch. ostensackenella* could have spread from Hokkaido to the Russian Far East. The dispersal of adult moths could be facilitated by wind currents or happen via transportation on boats circulating between Primorsky Krai and Japan. While the exact mechanisms and the distribution pathways of *Ch. ostensackenella* from Hokkaido to the Russian Far East remain speculative, the geographical proximity and potential transportation vectors could have contributed to the species’ introduction in the studied region.

The spread of *Ch. ostensackenella* in the Russian Far East is expected to be limited to Primorsky Krai, where its host plant, *R. pseudoacacia*, is grown in some man-made plantings. However, we also anticipate that multiple introductions of the moth may continue to occur from East Asian countries into Primorsky Krai. Considering the geographical proximity of China, Korea, and Japan, it is possible that ongoing introductions between these countries may continue to occur. Therefore, in the near future, the moth may occupy a significant part of East Asia where its host plant was introduced.

In China, the distribution of *R. pseudoacacia* is continuing to expand, and under climate change scenarios, it is expected to extend its secondary range northeastward [41]. It is likely that the invasive *Ch. ostensackenella* will respond to this and will follow its host plant. Likely, factors such as climate change, host plant distribution, and human-mediated introductions will continue to influence the spread of this invasive species.

Considering the recent recording of *Ch. ostensackenella* in Italy in 2022 (Figure 7) and the widespread distribution of North American *R. pseudoacacia* across Europe [40], the moth will likely continue spreading within the European continent. The insect’s distribution from European countries into European Russia in the near future is possible, as *R. pseudoacacia* is commonly planted in some urban areas of this part of Russia [43]. Given the significant damage caused by *Ch. ostensackenella* and *R. pseudoacacia* plantings in China [24], this species has the potential to become a notable pest of *R. pseudoacacia* in both East Asia and Europe. Furthermore, in Europe and the European part of Russia, *Ch. ostensackenella* may compete for trophic resource with two other invasive North American gracillariids associated with *R. pseudoacacia*, i.e., *Parectopa robiniella* and *Macrosaccus robiniella*. Early detection of *Ch. ostensackenella* is possible by examining *Robinia* leaves for the presence of mines whose characteristics were given in our study, and effectively distinguishing them from damage caused by these two outbreaking invasive species in Europe.

Lastly, the accidental introduction of *Ch. ostensackenella* to the Southern Hemisphere, where *R. pseudoacacia* was introduced [39], cannot be excluded. This highlights the importance of monitoring and preventive measures to minimize the potential spread of *Ch. ostensackenella* to new regions and mitigate its impact on *R. pseudoacacia* and invaded ecosystems.

## 5. Conclusions

Our study suggests that the occurrence of the North American *Ch. ostensackenella* in East Asia and Europe happened as two independent events, whereas its further expansion could be a result of dispersal from early infested locations in these continents. The detection of this moth species in the last seven years in three East Asian countries—China, South Korea, and Japan—and its recent discovery in the Russian Far East is the evidence of the continuous spread of *Ch. ostensackenella* in East Asia.

To gain a clearer understanding of *Ch. ostensackenella*’s long-distance jump from the New World to the Old World, more genetic data would be needed from both the moth’s natural range in North America and the invaded regions in Europe and East Asia.

The specific invasion routes of the species remain uncertain. While the possibility of accidental human-mediated introduction cannot be ruled out, further research is required to uncover the mechanisms behind the species introduction to new regions.

## Figures and Tables

**Table 1:** The specimens of *Chrysaster ostensackenella* sequenced from the Russian Far East and the DNA barcodes of this species borrowed from BOLD or GenBank for comparison.

No.	Sample ID	Country	Region, Place	Collection Date	Collectors ^1^	GenBank Accession Number
Original DNA barcodes						
1	NK1931	Russia	Primorsky Krai, MTS	02 August 2022	NAK	OR178285
2	NK1930	Russia	Primorsky Krai, Khorol	02 August 2022	NAK	OR178279
3	NK1929	Russia	Primorsky Krai, MTS	02 August 2022	NAK	OR178280
Borrowed DNA barcodes						
4	DNA-ATBI-3029	USA	Tennessee	19 May 2005	PH, J-FL	OR178278
5	DRD-05-0247	USA	Virginia	26 June 2005	JWB	KX069358
6	RMNH.INS.544312	USA	North Carolina	27 September 2010	EJN, CD	OR178282
7	RMNH.INS.18247	USA	North Carolina	27 September 2010	EJN, CD	OR178277
8	RMNH.INS.18245	USA	North Carolina	27 September 2010	EJN, CD	OR178281
9	CNCLEP00027381	USA	Tennessee	13 August 2006	JWB	OR178284
10	DNA-ATBI-3030	USA	Tennessee	19 May 2005	PH, J-FL	OR178276
11	BIOUG35791-H02	Canada	Ontario	09 June 2014	CBG	OR178283
12	TLMF Lep 31353	Italy	Abruzzen	31 June 2021	AM	OR178286
13	CO-SK1	South Korea	not indicated	06-07.2017	JMK, SKK, HEL	see [25]
14	MS044	Japan	Hokkaido, Mi	19 September 2021	MS, MSak	LC705457
15	MS043	Japan	Hokkaido, Mi	19 September 2021	MS, MSak	LC705456
16	MS042	Japan	Hokkaido, Mi	19 September 2021	MS, MSak	LC705455
17	MS041	Japan	Hokkaido, Mi	19 September 2021	MS, MSak	LC705454
18	MS036	Japan	Hokkaido, Uri	6 August 2021	MS, MSak	LC705453
19	MS035	Japan	Hokkaido, Uri	6 August 2021	MS, MSak	LC705452
20	MS034	Japan	Hokkaido, Uri	6 August 2021	MS, MSak	LC705451
21	MS033	Japan	Hokkaido, Uri	6 August 2021	MS, MSak	LC705450
Outgroup						
22	NK551	Russia	Primorsky Krai	23 June 2016	NIK	MK403724

^1^ Collectors: NAK—N.A. Kolyada; PH—P. Hebert; J-FL—J.-F. Landry; JWB—J.W. Brown; EJN—E.J. van Nieukerken; CD—C. Doorenweerd; AM—A. Mayr; JMK—J.M. Koo; SKK—S.K. Kim; HEL—H.E. Lee; MS—M. Sawada; MSak—M. Sakurai; CBG—CBG Collections Staff; NIK—N.I. Kirichenko; Mi—Minaminomori; Uri—Urimakunishi.

**Table 2:** Minimal and maximal intraspecific divergences in the COI mtDNA gene among the specimens of *Chrysaster ostensackenella* from different countries and region. Values in parentheses represent minimal to maximal genetic distances in each locality. Long dash (—) indicates no data because a single specimen was sequenced from a locality.

Country, Region ^1^	Country, Region							
	Russia, Primorsky Krai	Japan, Hokkaido	USA, North Carolina	South Korea	USA, Tennessee	USA, Virginia	Italy, Abruzzen	Canada, Ontario
Russia, Primorsky Krai	(0)							
Japan, Hokkaido	0–2.08	(0–2.08)						
USA, North Carolina	0.47–2.08	0–3.41	(1.86–3.29)					
South Korea	0.63	0–1.92	0.79–1.92	(—)				
USA, Tennessee	0.63–2.08	0.16–2.08	0–3.29	0.32–1.92	(0.16–1.92)			
USA, Virginia	0.63	0.32–1.92	0.46–1.92	0.32	0–1.92	(0)		
Italy, Abruzzen	0.95	0.32–1.92	0.79–1.92	0.32	0.32–1.92	0.32	(—)	
Canada, Ontario	2.08	0.32–2.08	0.33–3.08	1.92	0.32–1.92	1.92	1.60	(—)

^1^* Chrysaster ostensackenella* was represented by the following number of replications: Japan (8); Russia, Primorsky Krai (3); USA, North Carolina (3); USA, Tennessee (1); USA, Virginia (1); Canada, Ontario (1); Italy, Abruzzen (1); and South Korea (1).

**Table 3:** Presence of *Chrysaster ostensackenella* haplotypes in different countries in the Holarctic.

Haplotype	Specimens Process ID and Origin ^1^		Ratio
	Native Range	Invaded Range ^2^	
H1	—	LC705450|Uri|Japan OR178285|MTS|Russia OR178279|Khorol|Russia OR178280|MTS|Russia	0:4
H2	OR178282|North Carolina|USA	LC705455|Mi|Japan LC705451|Uri|Japan	1:2
H3	KX069358|Virginia|USA OR178276|Tennessee|USA	—	2:0
H4	OR178277|North Carolina|US	—	1:0
H5	—	LC705456|Mi|Japan LC705454|Mi|Japan LC705453|Uri|Japan S. Korea	0:4
H6	—	OR178286|Italy	0:1
H7	OR178283|Canada	—	1:0
H8	OR178284|Tennessee|USA	—	1:0
H9	OR178278|Tennessee|USA OR178281|North Carolina|USA	—	2:0
H10	—	LC705457|Mi|Japan LC705452|Uri|Japan	0:2
Sum of haplotypes ^3^	6	5	

^1^ Each specimen is indicated by GenBank accession number, region, and country; ^2^ MTS—Mountain-Taiga Station, Mi—Minaminomori, Uri—Urimakunishi; ^3^ One haplotype (H2) is shared.

## Data Availability

The genetic data used in the study are publicly accessible in BOLD using the link: http://doi.org/10.5883/DS-CHRYSORU (accessed on 16 May 2023).
